# Longitudinal changes in participant and informant reports of subjective cognitive complaints are associated with dementia risk

**DOI:** 10.3389/fnagi.2023.1044807

**Published:** 2023-02-20

**Authors:** Katya Numbers, Ben C. P. Lam, John D. Crawford, Nicole A. Kochan, Perminder S. Sachdev, Henry Brodaty

**Affiliations:** Centre for Healthy Brain Ageing (CHeBA), Discipline of Psychiatry and Mental Health, Faculty of Medicine and Health, University of New South Wales, Sydney, NSW, Australia

**Keywords:** subjective cognitive decline, ageing, dementia, study partner, longitudinal

## Abstract

**Background:**

Individuals with subjective cognitive complaints (SCCs) are at an increased risk of dementia. Questions remain about participant-reported versus informant-reported SCCs as indicators of future dementia and about longitudinal changes in participant-and informant-reported SCCs and risk of incident dementia.

**Method:**

Participants were 873 older adults (M = 78.65-years; 55% female) and 849 informants from the Sydney Memory and Ageing Study. Comprehensive assessments occurred biennially, and clinical diagnoses were made by expert consensus for 10-years. SCCs were participants’ and informants’ responses to a single binary question concerning their/the participant’s memory decline (Yes/No) over the first 6-years. Categorical latent growth curve analyses, using the logit transformation, were used to model SCC change over time. Associations of initial propensity to report SCCs at baseline, and change in propensity to report SCCs over time, with dementia risk were examined using Cox regression.

**Results:**

70% of participants reported SCCs at baseline, with a proportional increase in the odds of reporting by 11% for each additional year in the study. In contrast, 22% of informants reported SCCs at baseline, with a proportional increase by 30% in the odds of reporting per year. Participants’ initial level of (*p* = 0.007), but not change in SCC reporting (*p* = 0.179), was associated with risk of dementia controlling for all covariates. Both informants’ initial level of (*p* < 0.001), and change in (*p* < 0.001), SCCs significantly predicted incident dementia. When modelled together, informants’ initial level of, and change in, SCCs were still independently associated with increased dementia risk (*p’s* < 0.001).

**Conclusion:**

These data suggest that informants’ initial impressions, and increased reporting, of SCCs appear to be uniquely prognostic of future dementia compared to participants’, even based on a single SCC question.

## Introduction

Subjective cognitive complaints (SCCs), or a self-reported change in cognitive ability, are currently a core criterion for a diagnosis of Mild Cognitive Impairment (MCI) and Alzheimer’s disease (AD; Petersen, 2004; Dubois et al., 2007). Increasingly, SCCs are considered to be the earliest detectable prodromal stage of AD (Sperling et al., 2011; Mitchell et al., 2014) and have been linked to the presence of AD biomarkers like amyloid plaques in the brain (Jack et al., 2013), tau proteins in cerebral spinal fluid (Amariglio et al., 2015), and cerebral atrophy and/or hypometabolism (Mosconi et al., 2008; Striepens et al., 2010). Despite the potential relevance of SCCs in diagnosing a pre-clinical stage of AD, results are still mixed regarding the association between SCCs and objective cognitive performance and their efficacy for identifying individuals at greater risk of cognitive decline or incident dementia (e.g., Purser et al., 2006; Edmonds et al., 2014; Burmester et al., 2016; Hertzog et al., 2018). A possible explanation for these differences in results could be the well-established relationships of mood (e.g., depression and anxiety) and certain personality traits (e.g., neuroticism and conscientiousness) with SCCs (Comijs et al., 2002; Duchek et al., 2007; Buckley et al., 2013), with some researchers arguing that self-reported SCCs are better considered a proxy for underlying tendencies towards stress and rumination (Edmonds et al., 2014).

Another complicating factor is whether an individual can accurately acknowledge and report on cognitive changes as self-reports can be biased by both under-and overreporting of changes throughout the disease continuum (Ryu et al., 2020). A further complication is that the rate of SCC is strongly influenced by age such that the proportion of SCC in those aged under 65 is about 20%, but rises to about 90% in those over 85 (Bassett and Folstein, 1993), making it difficult to discriminate SCCs associated with normal cognitive ageing from SCCs due to early dementia pathology (Mitchell, 2008). Thus, attention has turned towards exploring the utility of informant-reported SCCs (i.e., SCCs regarding the participant’s cognitive ability) with some studies showing that informant-reports may be better indicators of current cognitive ability than self-reports (Rabin et al., 2012; Gifford et al., 2015; Nicholas et al., 2017; Ryu et al., 2020) and are more strongly associated with future cognitive decline and risk of dementia (Gifford et al., 2014, 2015; Numbers et al., 2020). Additional research (Rueda et al., 2015) has shown that informant-reported SCCs significantly correlate with Aβ42 and tau cerebral spinal fluid (CSF) levels and smaller hippocampal volumes, and that these associations are stronger than those with self-reported SCCs (see, too, Okonkwo et al., 2010). For these reasons, confirmation of cognitive changes from an informant is now a key SCD-*plus* criterion – a feature of SCC that increases the likelihood of preclinical AD – as outlined by the Subjective Cognitive Decline Initiative (SCD-I; Jessen et al., 2014).

Associations between changes in SCCs, as captured by a continuous measure, and changes in objective cognitive performance have been investigated by several researchers over the years (Jorm et al., 2001; Zimprich et al., 2003; Parisi et al., 2011). Recently, our group modelled changes in participant-reported complaints over 6 years using a continuous measure of SCCs (Numbers et al., 2021). We found that participants who reported increasing SCCs over 6 years had a steeper decline in global cognition in the same period of time and a nearly 4-fold increased risk of dementia over 10-years. Together these studies, which utilize a variety of modelling techniques to interrogate continuous SCC data, have shown that consistently and increasingly reported SCCs predict cognitive changes over time. Other studies (Roehr et al., 2016; Wolfsgruber et al., 2016) have also investigated the associations between longitudinal characteristics of a *single* SCC question and incident MCI and/or dementia by examining the stability of the SCC response (i.e., ‘stable’ vs. ‘not stable’) only. Both found that individuals who consistently reported SCC at each follow-up (‘Do you feel like your memory is becoming worse?’ yes/no) were more likely to progress to MCI and dementia. To our knowledge, no study has investigated longitudinal change in a binary measure of SCCs as a predictor of incident dementia.

By the time individuals with subjective complaints receive a clinical diagnosis, informant reports are often used to validate the individual’s concern, but in the very early stages of MCI and dementia it is unclear whether it is the individual or the informant that is better able to provide a more accurate evaluation of cognitive changes. Determining the utility of observing changes in participant-and informant-reported SCCs over time has clinical applications as this type of information can be gathered quickly and efficiently compared to other screening tools for dementia. Although previous studies have examined the association between informant-reported SCCs and various outcomes related to cognitive decline and risk of dementia, to our knowledge, no study has modelled and compared longitudinal changes in both participant-and informant-reported SCCs – as captured by a single yes/no question – and the associated risk of dementia. Thus, the present study aims to interrogate the longitudinal properties of a binary SCC response option to determine the extent to which participants’ and informants’ propensity to endorse SCC at baseline, and their change in propensity to endorse SCC over time, predict increased risk of dementia.

## Methods

### Participants

Community-dwelling older adults aged 70–90 years, living in the Eastern Suburbs of Sydney, Australia, were selected *via* the electoral roll and invited to participate in the Sydney Memory and Ageing Study (MAS) in 2005. Of the 8,914 individuals invited to participate, 1,037 participants were included in the baseline sample. Inclusion criteria were the ability to speak and write English sufficiently well to complete a psychometric assessment and self-report questionnaires. Exclusion criteria included any major psychiatric diagnoses, acute psychotic symptoms, or a current diagnosis of multiple sclerosis, motor neuron disease, developmental disability, progressive malignancy, or dementia. More detailed methods of recruitment and baseline demographics have been previously described by Sachdev et al. (2010). For the current study, participants were further excluded if they were not able to speak English at a basic conversational level by the age of 9 (*N* = 164) because of the questionable validity of using normative data based on persons of English-speaking background to determine cognitive impairment in these individuals (Kochan et al., 2010).

Of the 873 participants included in the present study, 849 (97.3%) had an informant at baseline. Informants were nominated by the participants and answered questions relating to the participant’s memory, thinking, and daily functioning at each study wave. Informants were required to have at least 1 h of contact with the participant per week and were composed of spouses, children, family members, and close friends or contacts.

**Figure 1:**
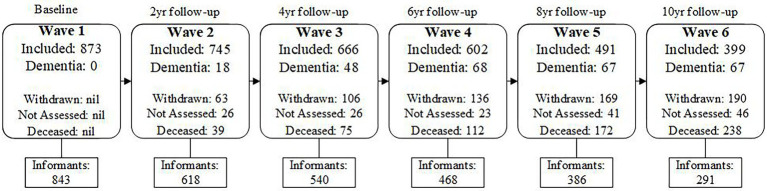
Flow chart of Ns for included participants and informants, incidence of dementia, and Participant attrition due to withdrawing or death across Waves 1–6.

Comprehensive assessments consisting of medical history, medical examination, neuropsychological testing, and subjective cognitive complaints were conducted by trained research assistants at two-yearly intervals (called waves). At each wave, informants completed comprehensive telephone interviews and questionnaire packets about themselves and the participants. All participants and informants provided written consent to participate in this study, which was approved by the University of New South Wales Human Ethics Review Committee (HC: 05037, 09382, 14327). A study flowchart outlining participant and informant sample size, dementia incidence and participants’ reason for attrition at each wave is presented in Figure 1.

### Subjective cognitive complaints

At each of the first 4 waves, participants were asked to respond to the question *“Have you noticed difficulties with your memory? - yes/no,”* and informants were asked to respond to the question *“Have you noticed the participant having difficulty with their memory? – yes/no.”* At baseline (Wave 1), the preceding question stem read: “*In the last* ***5 years*:**”; and in all subsequent waves, the preceding question stem read “*In the last **2 years***:.” This change was made to capture participants’ and informants’ perceptions of memory decline during the time between assessments. At each wave, a “yes” response was scored as 1 and a “no” response was scored as 0.

### Consensus diagnosis

Clinical diagnoses were available for Waves 1 to 6 (10-year follow-up). At baseline, and at each two-year follow-up, participants who met a range of clinical criteria were brought to a consensus review meeting where at least three clinicians from a panel of neuropsychiatrists, psychogeriatricians, and neuropsychologists discussed all available clinical, neuropsychological, blood chemistry and imaging data to reach a consensus diagnosis. Cases were brought to consensus review under the following conditions: impaired performance on neuropsychological tests (at least 1.5 SDs below published normative data on two cognitive measures), impaired informant-reported independent activities of daily living (IADLs; Hindmarch et al., 1998), and neuropsychiatric symptoms. Participants who did not meet the conditions for referral to the consensus panel were coded as “not dementia” for each wave.

MCI was diagnosed using international consensus criteria (Winblad et al., 2004). A diagnosis of MCI was made if *all* of the following criteria were met: (a) complaint of decline in memory or other cognitive function which may be self or informant-reported; (b) cognitive impairment on objective testing that was not normal for age as determined by performance on at least one test score of 1.5 SDs or more *below* published normative values (or comparable standardized score provided in the normative source compared to age and/or education-matched samples); (c) not demented – participants did not have a pre-existing diagnosis of dementia on entry to the study, had an adjusted MMSE score of ≥24 and did not meet DSM-IV criteria for possible or probable dementia; (d) essentially normal function or minimal impairment in instrumental activities of daily living (IADLs) defined by a total average score < 3.0 on the Bayer ADL Scale (Hindmarch et al., 1998).

A diagnosis of dementia was based on the Diagnostic and Statistical Manual of Mental Disorders, Fourth Edition (DSM-IV; American Psychiatric Association, 2013)—that is, impairment in the cognitive domain of memory, plus impairment in one other cognitive domain that is sufficiently severe as to cause impairment in functioning (Bayer IADL scale score ≥ 3.0; Hindmarch et al., 1998). Although cut-offs were used, descriptive narratives, clinical observations, and diagnoses of dementia made by external medical specialists were also considered when making a diagnosis. Individuals who did not receive a dementia diagnosis were classified as “not dementia” at each wave, and no dementia cases were present at baseline as this was an exclusionary criterion.

### Covariates

Demographic information, including age, sex, education, and native-English speaking status, were collected at baseline APOE*4 status was determined using genomic DNA that was extracted from peripheral blood leukocytes; genotyping was performed on the two single nucleotide polymorphisms (rs7412 and rs429358) that distinguish between the three *APOE* alleles ε2, ε3, and ε4 using Taqman assay. Depression and anxiety were measured by the 15-item version of the Geriatric Depression Scale (GDS; Sheikh and Yesavage, 1986) and the Goldberg Anxiety Scale (GAS; Goldberg et al., 1988), respectively. Neuroticism, Openness, and Conscientiousness scales of the NEO-Five Factor Inventory (NEO-FFI) were administered (Costa and McCrae, 1989). Informant’s age, sex, length of relationship with participant (years), and type of relationship with participant were collected.

### Statistical analyses

Latent growth curve modelling (LGCM) was conducted using structural equation modelling (SEM) to model the trajectories of participant and informant SCCs over 6 years. The conventional approach of LGCM is applied to continuous variables, while its application to categorical variables requires the use of latent variable transformation, like that used in generalized linear modelling (Feldman et al., 2009; Masyn, 2013; Lee et al., 2018). Logit transformation, as in logistic regression, is used to model binary outcome variables. With this approach, the probability of the observed binary variable was transformed to odds, and then to log-odds (i.e., logit), with a standardized logistic distribution, representing an individual’s level in a continuous latent variable. Latent growth curve analysis was then conducted on these latent continuous variables in a similar manner to observed continuous variables, with one major difference being that in conventional LGCM, the latent intercept and slope variables are in the same unit of the observed continuous variables, but in categorical LGCM, the intercept and slope are in logit values, requiring conversion to odds and odds ratio, respectively, for easier interpretation.

**Figure 2:**
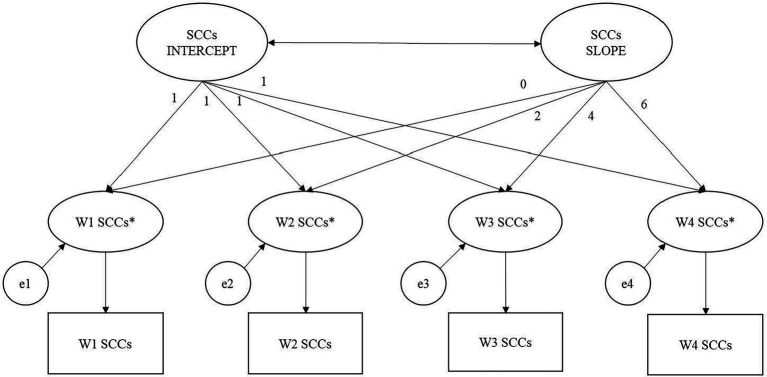
Categorical latent growth model of subjective cognitive complaints (SCCs). Note. Squares represent observed binary SCCs variables over 4 waves. Asterisks represent continuous latent response variable transformed from binary SCCs variable using logit transformation e1–e4 are residuals of the latent response variable. The factor loading for the latent intercept are set to 1, while those for the latent linear slope are set to 0, 2, 4, and 6, respectively, to reflect the spacing of time point. Thresholds of the binary SCCs variables are fixed at 0 for model identification and for estimating the mean of the latent intercept factor. W1 = baseline (Wave 1); W2 = 2-year follow-up (Wave 2); W3 = 4-yar follow-up (Wave 3), W4 = 6-year follow-up (Wave 4).

Latent growth factors of intercept and slope were based on the four measurements of binary SCCs collected at Waves 1–4. The factor loadings for the latent intercept were set to 1, while those for the latent linear slope were set to 0, 2, 4, and 6, respectively, to reflect the spacing of time points. Hence, the intercept represents the initial log-odds of reporting a SCC, and the slope represents the linear change in the log-odds of reporting a SCC per year. Thresholds of the binary variables (i.e., cut-points on the continuous latent response variables used to define the binary SCC variables) were fixed at 0 for model identification and for estimating the mean of the intercept factor, following conventional practice (Mehta et al., 2004). In the first set of analyses, the individual trajectories of participant and informant SCCs over time was estimated in separate unconditional LGCMs with a latent intercept and a latent linear slope (see Figure 2). Models adding a latent quadratic slope were also examined and compared with the first models using Akaike Information Criteria (AIC) and Bayesian Information Criteria (BIC). Then, a parallel process model was run to examine the associations between participants’ and informants’ initial levels of (intercept), and changes in (slope), SCCs over 6 years.

In the last set of analyses, Cox proportional hazards regression were conducted to examine the associations of the initial levels of, and changes in, participant and informant SCCs and the risk of dementia over a 10-year period, as clinical diagnoses were available across all 6 waves. Time to progression to dementia was specified at the midway point between the assessment that dementia was first diagnosed and the previous assessment, while event times were censored at the end of follow-up or participant drop-out. We first examined the effects of participant and informant SCCs in two separate models before entering them together in the same model to investigate their contributions to risk of dementia. It is worth noting that a Hazard Ratios (HR) greater than 1 indicates an increase in risk of dementia per one unit increase in a predictor. Because the SCC intercept and slope were in logit values, we standardized them against the sample average for easier interpretation, such that a one-unit change represented one standard deviation change in intercept and slope in logit values in the sample.

Each set of Cox regressions was adjusted for participants’ age, sex, education, *APOE4* status, GDS, GAS, and neuroticism, openness, and consciousness scores at baseline. For the individual informant model, we ran two sets of Cox regressions. The first controlled for all covariates listed above and the second controlled for the additional covariates of informants’ age, sex, education, type of relationship with the participant (spouse vs. grand/child vs. other family members vs. friends and others), and length of relationship with participants (in years). It is worth noting that none of the additional informant covariates were significantly associated with risk of dementia and results were similar (see Supplementary Table S1), and thus these informant covariates were not included in the final model, which examined both participants’ and informants’ SCCs concurrently, still controlling for all participant covariates.

Three sets of sensitivity analysis based on our final model were conducted. First, we examined a cause-specific hazard model for dementia diagnosis accounting for death. In this model, censoring was specified on the date of death or at the end of follow-up/participant drop-out. The second, analysis was conducted using Wave 4 (6-year follow-up) as the new baseline to examine progression to dementia over a 4-year period, given that the SCC intercept and slope were estimated using data from Wave 1 to Wave 4. Finally, we adjusted for baseline MCI status given the established relationship between MCI and dementia risk (Petersen, 2004). We also explored whether the associations between SCC intercept and slope and dementia risk would differ among people with normal cognition and MCI diagnosis by incorporating interaction terms with MCI status in the model.

Data were prepared and basic statistics were computed using the Statistics Package for the Social Sciences (SPSS) version 23. Structural equation modelling with maximum likelihood estimation with robust standard errors, for modelling non-Gaussian distributions of the variables, was conducted using Mplus version 6 (Muthén and Muthén, 2017). Full information maximum likelihood (FIML) was used to reduce bias due to non-random missing data. Results were regarded as significant if *p* < 0.05.

## Results

### Sample characteristics

Basic descriptive statistics for baseline participants and informants are presented in Table 1. Participants were on average 78.65 (SD = 4.79) years old and were well educated (M = 11.62 years, SD = 3.50) for their birth cohort. At study entry, 56% were female and 23% tested positive for the *APOE4*allele. In general, participants did not meet clinical criteria for anxiety (i.e., GAS score ≥ 4) or depression (i.e., GDS score ≥ 5; Jessen, 2014; Jessen et al., 2020). At baseline, 38.8% of the participants were diagnosed with MCI. Informants were on average 62. Ninety three (SD = 13.91) years old at baseline, and 68% were female. On average, informants had known participants for 45 years and approximately 38% reported living with the participant. Regarding their relationships with the participants, 30% were spouse, 37% were a child or grandchild, 9% were other family members, and 25% were friends and others. The proportion of participants reporting SCCs at each wave were: Wave 1 = 65%, Wave 2 = 61%, Wave 3 = 64%, and Wave 4 = 74%. For informant SCCs, the proportions were much lower across waves at: Wave 1 = 35%, Wave 2 = 42%, Wave 3 = 43%, and Wave 4 = 54%.

**Table 1:** Basic descriptive, including mean, standard deviation (or N and %) for all predictor variables used in the analyses.

	*M* (*SD*) or *N* (%)	Range
Participant demographics – *N* = 873		
Age in years	78.65 (4.79)	70–91
No. of Women (*N*, %)	490 (56.1)	-
Education in years	11.62 (3.50)	3–24
GDS score	2.21 (1.99)	0–14
GAS score	1.11 (1.89)	0–8
Neuroticism	15. 13 (7.04)	0–39
Conscientiousness	33.86 (6.05)	13–48
Openness	26.89 (6.02)	10–43
*APOE4* carrier (*N*, %)	191 (23.1)	
Baseline SCC – “yes” (N, %)	519 (65.0)	-
Informant demographics *N* = 849		
Age in years	62.93 (13.91)	24–95
No. of Women (*N*, %)	578 (68.1)	-
Years known to participant	45.35 (15.94)	2–84
Relationship to participant (*N*, %)		
Spouse	251 (29.6)	-
Child/grandchild	313 (36.9)	-
Other family member	77 (9.1)	-
Friend/other	208 (24.5)	-
Baseline SCC – “yes” (*N*, %)	218 (34.5)	

GDS = geriatric depression scale; GAS = Goldberg anxiety scale; neuroticism, contentiousness and openness scores are captured *via* the NEO-five factor inventory; Baseline SCC = N (%) of participants/informants that responded yes to the single question “Have you noticed changes in your/the participant’s memory in the last 5 years?”

### Trajectories of subjective cognitive complaints

Results from the unconditional growth curve models estimated that initially about 70% of the participants reported SCCs (Odds = 2.36, *p* < 0.001, *SD* = 9.67). There was a proportional increase of 11% per year in the odds of reporting SCCs by participants (Odds ratio = 1.11, *p* = 0.001, *SD* = 1.21). In contrast, the model estimated that only 22% of the informants reported SCCs initially (Odds = 0.29, *p* < 0.001, *SD* = 12.71). However, there was a proportional increase of 30% per year in the odds of reporting SCCs by informants (OR = 1.30, *p* < 0.001, *SD* = 1.43). Adding a quadratic slope in both models did not improve fit based on comparing AIC and BIC, and hence was not considered in subsequent analyses.

Pearson’s correlations revealed the initial levels of participant and informant SCCs were positively correlated (*r* = 0.32, *p* < 0.001), while the slopes were not (*r* = −0.03, *p* = 0.34). Consistent with these findings, the parallel process model revealed that, after controlling for the participant covariates, the initial levels of participant and informant SCCs were significantly associated (covariance = 2.49, *p* < 0.001; standardized estimate = 0.51),^1^ while participant and informant SCC slopes were not (covariance = 0.03, *p* = 0.158; standardized estimate = 0.48). Controlling for informant covariates did not change the patterns of results.

### Subjective cognitive complaints and risk of dementia

Table 2 presents the results of the three Cox regression analyses examining standardized participant and informant SCCs predictors and risk of incident dementia, controlling for all covariates (for results using unstandardized SCCs predictors, see Supplementary Table S2). At baseline, after controlling for all covariates, there was a significant positive association between participants’ SCC intercept and incident dementia (Hazard ratio = 1.25, *p* = 0.007); however, participants’ SCC slope was not significantly associated with risk of dementia (HR = 1.12, *p* = 0.179). For informants, controlling for all covariates, there was a significant positive association between SCC intercept and risk of dementia (HR = 1.57, *p* < 0.001), as well as SCC slope (HR = 1.37, *p* < 0.001).

**Table 2:** Results of Cox proportional hazard regression models predicting incident dementia over 10 years for participants (Model 1), informants (Model 2) and both participants and informants (Model 3), controlling for participants’ baseline demographics, ApoE4 carrier status, mood, and personality.

	Model 1 participant SCCs				Model 2 informant SCCs				Model 3 participant & informant SCCs			
		95% CI				95% CI				95% CI		
^†^Predictors	HR	LL	UL	*p*	HR	LL	UL	*p*	HR	LL	UL	*p*
^†^Participant SCC intercept	1.25	1.06	1.47	**0.007**					1.02	0.85	1.21	0.858
^†^Participant SCC slope	1.12	0.95	1.31	0.179					1.08	0.92	1.27	0.032
^†^Informant SCC intercept					1.57	1.30	1.89	**<0.001**	1.57	1.29	1.89	**<0.001**
^†^Informant SCC slope					1.37	1.14	1.63	**0.001**	1.36	1.13	1.63	**0.001**
Age	1.12	1.09	1.16	**<0.001**	1.12	1.08	1.15	**<0.001**	1.12	1.08	1.15	**<0.001**
Sex	0.82	0.60	1.14	0.236	0.95	0.68	1.34	0.774	0.94	0.67	1.32	0.720
Education	1.04	1.00	1.09	0.065	1.03	0.99	1.08	0.139	1.04	0.99	1.08	0.124
*APOE4* status	2.01	1.49	2.71	**<0.001**	1.83	1.33	2.50	**<0.001**	1.79	1.30	2.45	**<0.001**
GDS	0.82	0.88	1.09	0.703	0.95	0.85	1.06	0.349	0.95	0.85	1.06	0.385
GAS	1.09	0.96	1.13	0.373	1.01	0.93	1.10	0.753	1.01	0.93	1.10	0.811
Neuroticism	0.97	0.96	1.01	0.349	0.99	0.96	1.02	0.357	0.99	0.96	1.02	0.379
Openness	0.97	0.95	1.00	0.053	0.97	0.95	1.00	0.072	0.97	0.95	1.00	0.072
Consciousness	0.99	0.97	1.02	0.0681	0.099	0.97	1.22	0.573	0.99	0.97	1.02	0.613

GDS = geriatric depression scale; GAS = Goldberg anxiety scale; neuroticism, contentiousness and openness scores are captured *via* the NEO-five factor inventory.

† SCC intercept and slope for participants and informants are standardized against the sample average. Bold = *p* < 0.05.

When participant SCCs entered into the model with informant SCCs, still controlling for all covariates, participants’ SCC intercept became non-significant (HR = 1.02, *p* = 0.858) and participants’ slope remained non-significant (HR = 1.08, *p* = 0.329). In contrast, after controlling for all covariates, both informants’ SCC intercept and slope remained significant (HR = 1.57, *p* < 0.001, and HR = 1.36, *p* < 0.001, respectively). Among the covariates, older age (HR = 1.12, *p* < 0.001) and ApoE4 status (HR = 1.79, *p* < 0.001) were also significantly associated with increased risk of dementia. The informant covariates were not significantly associated with dementia risk and controlling for them did not change the patterns of results.

Cause-specific hazard model accounting for the competing risk of death revealed significant associations between informants’ SCC intercept and slope and increased risk of dementia (HR = 1.61, *p* < 0.001, and HR = 1.38, *p* < 0.001, respectively; see Supplementary Table S3). These effects were comparable to those observed in the main analysis.

Informants’ SCC intercept remained significantly associated with increased risk of dementia over a 4-year period (HR = 1.35, *p* = 0.019) when using Wave 4 as the baseline (see Supplementary Table S4). However, informants’ SCC slope was not significant (HR = 1.23, *p* = 0.113), and participants’ SCC intercept and slope were also non-significant.

When adjusting for baseline MCI status, both informants’ SCC intercept and slope were again significantly associated with increased dementia risk with comparable effects to the main analyses (HR = 1.42, *p* = 0.001, and HR = 1.36, *p* = 0.002, respectively; see Supplementary Table S5). Again, participants’ SCC intercept and slope were non-significant, and no significant interaction effect between MCI status and SCC intercept and slope was observed.

### Discussion

Increasingly, evidence from epidemiological and biomarker studies suggest that SCCs are an early indicator of an early, pre-MCI stage of AD known as SCD (Jack et al., 2013; Jessen, 2014; Jessen et al., 2014, 2020). Research is therefore increasingly focused on capturing the qualitative characteristics of SCD that are most predictive of increased likelihood of underlying pre-clinical AD in cognitively normal individuals. Persistence of SCC over time (Roehr et al., 2016; Wolfsgruber et al., 2016; van Harten et al., 2018), and confirmation of cognitive decline by an observer (Valech et al., 2015; Numbers et al., 2020; Ryu et al., 2020), are repeatedly associated with increased likelihood of cognitive decline and incident dementia. For this reason, persistence and confirmation are classed as SCD-*plus* features, or SCC features that increase the risk of future objective cognitive decline (Jessen et al., 2014, 2020). In the present study, we examined longitudinal change in participant and informant reports of SCC over 6 years, to determine whether these SCC features were associated with increased risk of incident dementia over 10 years. To do so, we modelled linear change in participants’ and informants’ propensity to report SCC over time using categorical latent growth curve models to estimate within-person changes (i.e., slopes) and between-person differences in change (i.e., variation in slopes) for participants and informants over 6 years.

At baseline, over 70% of participants endorsed SCC compared to only 23% of informants. However, over 6-years, participants’ propensity to report SCC increased proportionally by 11% compared to 30% in propensity to report SCCs for informants. Our results provide further evidence that examining SCC longitudinally, and collecting informant corroborations of SCC over time, provide valuable information about individuals more at risk of progressing to dementia.

When modelled individually, participants’ baseline propensity to report SCCs was significantly associated with increased risk of dementia, though the modelled change in SCC reporting over 6 years was not. For one standard deviation difference from the average log odd values in participants’ initial reporting of SCCs, risk of dementia increased by 16%. When modelled individually, informants’ initial propensity to report SCCs, and change in SCCs reporting over 6 years, were both significantly associated with increased risk of dementia. In fully adjusted models, for a one standard deviation difference from the average log odd values in the informants’ initial reporting of SCCs, there was a 57% increase in risk of dementia. For one standard deviation difference from the average log odd values in change of reporting SCCs per year by informants, there was a 37% increase in the risk of developing dementia.

Next, when modelling participant and informant SCCs together, informants’ initial impressions of SCCs, and increased propensity to switch to reporting SCCs, appear to be uniquely prognostic of future dementia compared to participants’ initial propensity, and change in propensity, to report SCCs. That is, controlling for participant SCCs and all relevant covariates, a one standard deviation difference from the average log odd values in change of reporting SCCs per year by informants was associated with a 36% increased risk of incident dementia over 10 years. Sensitivity analyses adjusting for the competing risk of death, as well as baseline MCI status revealed similar patterns of results. While informants’ change in propensity to report SCCs was not significantly associated with dementia risk over the 4-year period after SCC change was estimated, this observation could potentially be biased by the smaller sample used, selective sampling of healthier survivors, and shorter follow-up. Taken together, these findings are in line with the updated SCD-*plus* framework (Jessen et al., 2020) and other studies suggesting that informant-reported SCCs are associated with an increased likelihood of future cognitive decline (Caselli et al., 2014; Valech et al., 2015; Nicholas et al., 2017). Our findings that participants’ initial reporting of SCC, but not change in propensity to report SCCs, was associated with increased risk of dementia aligns with other studies that have shown individuals with SCD will only experience and accurately report on cognitive decline at the very early stages of the disease, whereas observers will more accurately report on mild cognitive changes and dysfunctions at more advanced stages of cognitive decline (Caselli et al., 2014; Nicholas et al., 2017).

And though our investigation into longitudinal stability of SCCs does not directly replicate the methodology of studies (Roehr et al., 2016; Wolfsgruber et al., 2016) cited in the updated SCD-*plus* framework presented by Jessen and colleagues (Jessen et al., 2020), it adds to the discussion around temporal characteristics of SCCs that are most associated with increased risk of dementia. Namely, that propensity to endorse SCCs over time, especially by a knowledgeable informant, is highly indicative of increased risk of incident dementia, even above well-established genetic determinants like *APOE4* carrier status. Additionally, our result compliment earlier research examining linear change in continuous SCC measures and increased rates of decline in memory and global cognition (Jorm et al., 2001; Zimprich et al., 2003; Parisi et al., 2011) and other cognitive domains (Snitz et al., 2015) and are in line with recent research from our own group showing participants’ initial reporting of SCCs-captured by a continuous measure - predict increased risk of incident dementia. Taken together, these results suggest that investigating longitudinal change in SCCs – especially those provided by an informant – can provide important information about individuals at the greatest risk of developing dementia and may be a useful future SCD-*plus* feature.

Our results should be considered within the context of several limitations. First, for our results to have clinical relevance or to be applicable to researchers, SCC data from multiple time points must be available as the modelling techniques we employ here cannot be performed on data from a single occasion, nor can the model be applied to a single individual. Further, according to the data presented here, patients must present with a knowledgeable informant on multiple occasions who can comment on their cognitive changes. Thus, for clinicians, an ongoing relationship with the patient *and* an informant would be required to detect change in SCCs reporting, as informant reports appear to be more indicative of increased risk of dementia compared to participant reports.

Second, we operationalized SCC categorically using only a single “Yes/No” question. To do this, we used latent growth curve models, which are conventionally applied to continuous variables where the residuals are assumed to be normally distributed. Because categorical variables (i.e., binary and ordinal variables) do not hold to normal distribution assumptions, our analyses required an additional step of transforming observed categorical responses into latent continuous variables. This can make meaningful interpretations of effects sizes, at least in their original units, challenging. Further, as other studies have noted (Roehr et al., 2016; Wolfsgruber et al., 2016), it is possible that a single SCC question lacks the ability to capture more fine-grained change in SCC over time compared to a continuous measure of subjective decline. And although our group (Numbers et al., 2021) has reported a similar pattern of results when modelling participant and informant SCCs captured by a continuous measure (i.e., the MAC-Q), more research in this area is required to determine the clinical significance of temporal changes in SCC, especially as changes in a continuous scale can be applied to an individual where these analyses can only be completed at a group level.

Finally, we did not interrogate different subtypes of either MCI (e.g., amnestic vs. non-amnestic, single vs. multi-domain) or dementia (e.g., AD, vascular, Lewy body, mixed) as these data were not available at every wave and the relatively small N associated with some subtypes (e.g., non-amnestic multi-domain MCI or Lewy body dementia). Future research could examine whether increasing propensity to report SCCs, gathered from either participants or informants, is more indicative of risk to certain kinds of MCI and/or dementia.

Strengths of our study include a large, well-characterized sample with clinical diagnoses made by an expert consensus panel over a relatively long period of time (10-years). Further, using transformed latent continuous SCC variables allowed us to model linear trajectories of binary SCCs over time, which may provide information over and above an examining of SCC stability (e.g., “stable” vs. “not stable”) over time using a similar binary response. For example, participants classed as “not stable” might include those who originally do not report SCCs but later do, and conversely those who originally do report SCCs but later do not (and any combination thereof), despite the potential for different underlying aetiologies. We suggest that modelling longitudinal changes in SCCs over time may better reflect the progression of AD pathology, even reflecting loss of insight associated with disease progression for participants.

## Conclusion

This study models longitudinal trends in SCC reporting for both participants and informants and examines the relationship between change in SCC and incident dementia using novel statistical techniques. Overall, participants were much more likely to initially report SCC compared to informants, though informants’ propensity to report increasing SCC was significantly steeper compared to participants’ change in propensity of reporting. Participants with a standard deviation increase in initial SCC reporting had 16% increased risk of dementia compared to a 57% increased risk of dementia risk for a standard deviation increase in informants initial SCC reporting. Importantly, a one standard deviation increase in informants’ propensity to report SCC each year was associated with a 37% increased risk of incident dementia, even controlling for participants’ initial, and change in, propensity to report SCCs and all relevant covariates. These results add to the growing body of literature examining temporal properties of SCCs and provide further insight into the dynamic nature of participant and informant SCCs, particularly highlighting the importance of including informant reported SCCs over time.

## Data availability statement

The terms of consent for research participation stipulate that an individual’s data can only be shared outside of the MAS investigators group if the group has reviewed and approved the proposed secondary use of the data. This consent applies regardless of whether data has been de-identified. Access is mediated via a standardised request process managed by the CHeBA Research Bank, who can be contacted at ChebaData@unsw.edu.au, or via the first author’s contact details at k.numbers@unsw.edu.au.

## Ethics statement

The studies involving human participants were reviewed and approved by University of New South Wales Human Research Ethics Committee (HREC). The patients/participants provided their written informed consent to participate in this study.

## Author contributions

KN is the corresponding author and guarantor of this manuscript. KN designed this study, wrote this manuscript, assisted with analyses, incorporated all levels of feedback from co-authors, and prepared this manuscript for submission. BL contributed significantly to the writing of this manuscript and was responsible for statistical analyses and assisted with interpretation of results. JC also contributed significantly to the data analyses and interpretation of results. NK and PS provided statistical, neuropsychological, and medical guidance, and reviewed this manuscript and revisions. HB provided expert guidance and mentorship for all stages of this project and reviewed this manuscript and revisions. The project was funded by successful project grants secured by PS and HB. All authors contributed to the article and approved the submitted version.

## Funding

HB and PS received the following grants to support this work: Three National Health & Medical Research Council (NHMRC) of Australia Program Grants (ID350833, ID568969, and APP1093083; https://www.nhmrc.gov.au/funding).

## Conflict of interest

The authors declare that the research was conducted in the absence of any commercial or financial relationships that could be construed as a potential conflict of interest.

## Publisher’s note

All claims expressed in this article are solely those of the authors and do not necessarily represent those of their affiliated organizations, or those of the publisher, the editors and the reviewers. Any product that may be evaluated in this article, or claim that may be made by its manufacturer, is not guaranteed or endorsed by the publisher.

## Footnotes

^1^Residual covariance measures the total variability of two variables in their original units, adjusting for other variables predicting the two. Standardised estimate is computed by standardising the variances of the two variables.
