# Supplementary material for: Longitudinal changes in participant and informant reports of subjective cognitive complaints are associated with dementia risk
Source: Front Aging Neurosci. 2023 Feb 20;15:1044807. doi: 10.3389/fnagi.2023.1044807 (PMC9987247; doi:10.3389/fnagi.2023.1044807)
Supplement: Supplementary file 1 [file Table_1.docx]

**Supplementary Table 1.** Results of fully adjusted Cox proportional hazard regression models predicting incident dementia over 10 years for informants, controlling for participants’ baseline demographics, *APOE4* carrier status, mood, personality, informant age, informant sex, years known to participant, and relationship to participant (child, spouse/family, friend/other) using standardised SCC predictors.

|  | Informant SCCs | | | | | |
| --- | --- | --- | --- | --- | --- | --- |
|  |  | | 95% CI | |  | |
| Predictors | HR | LL | | UL | | *p* |
| †Informant SCC intercept | 1.47 | 1.18 | | 1.82 | | **.001** |
| †Informant SCC slope | 1.49 | 1.21 | | 1.85 | | **< .001** |
| Age (Participant) | 1.13 | 1.08 | | 1.19 | | **< .001** |
| Sex (Participant) | 0.96 | 0.62 | | 1.49 | | .870 |
| Education (Participant) | 1.05 | 1.00 | | 1.11 | | .055 |
| *APOE4* status | 1.64 | 1.12 | | 2.40 | | **.011** |
| GDS | 0.90 | 0.79 | | 1.02 | | .097 |
| GAS | 0.98 | 0.88 | | 1.09 | | .710 |
| Neuroticism | 1.01 | 0.98 | | 1.05 | | .504 |
| Openness | 0.97 | 0.93 | | 1.00 | | .066 |
| Consciousness | 1.00 | 0.96 | | 1.03 | | .802 |
| Informant Age | 0.98 | 0.96 | | 1.01 | | .212 |
| Informant Sex | 1.02 | 0.69 | | 1.49 | | .934 |
| Years Know to Participant | 1.00 | 0.98 | | 1.01 | | .874 |
| Relationship to Participant  Child  Spouse/Family Member  Friend/Other | 0.61  1.22  0.97 | 0.27  0.49  0.56 | | 1.41  3.02  1.68 | | .250  .671  .927 |

Note: GDS = Geriatric Depression Scale; GAS = Goldberg Anxiety Scale; Neuroticism, Contentiousness and Openness scores are captured via the NEO-Five Factor Inventory.

†SCC intercept and slope are standardized against the sample average.
