# Supplementary material for: Longitudinal changes in participant and informant reports of subjective cognitive complaints are associated with dementia risk
Source: Front Aging Neurosci. 2023 Feb 20;15:1044807. doi: 10.3389/fnagi.2023.1044807 (PMC9987247; doi:10.3389/fnagi.2023.1044807)
Supplement: Supplementary file 2 [file Table_2.docx]

**Supp Table 2.** Results of Cox proportional hazard regression models predicting incident dementia over 10 years for participants (Model 1), informants (Model 2) and both participants and informants (Model 3), controlling for participants’ baseline demographics, *APOE4* carrier status, mood, and personality using *unstandardised SCC predictors* (i.e., slope and intercept).

|  | Model 1  Participant SCCs | | | | | | Model 2  Informant SCCs | | | | | | Model 3  Participant & Informant SCCs | | | | | |
| --- | --- | --- | --- | --- | --- | --- | --- | --- | --- | --- | --- | --- | --- | --- | --- | --- | --- | --- |
|  |  | | 95% CI | |  | |  | | 95% CI | |  | |  | | 95% CI | |  | |
| Predictors | HR | LL | | UL | | *p* | HR | LL | | UL | | *p* | HR | LL | | UL | | *p* |
| Participant SCC intercept | 1.13 | 0.04 | | 0.22 | | **.007** |  |  | |  | |  | 1.01 | 0.91 | | 1.12 | | .858 |
| Participant SCC slope | 9.16 | -1.02 | | 5.45 | | .179 |  |  | |  | |  | 5.04 | 0.20 | | 130.32 | | .329 |
| Informant SCC intercept |  |  | |  | |  | 1.26 | 1.15 | | 1.39 | | **< .001** | 1.26 | 1.14 | | 1.39 | | **< .001** |
| Informant SCC slope |  |  | |  | |  | 6.99 | 2.28 | | 21.41 | | **.001** | 6.78 | 2.15 | | 21.37 | | .001 |
| Age | 1.12 | 0.09 | | 0.15 | | **< .001** | 1.12 | 1.08 | | 1.15 | | **< .001** | 1.12 | 1.08 | | 1.15 | | **< .001** |
| Sex | 0.82 | -0.52 | | 0.13 | | .236 | 0.95 | 0.68 | | 1.34 | | .774 | 0.94 | 0.67 | | 1.32 | | .720 |
| Education | 1.04 | -0.00 | | 0.09 | | .065 | 1.03 | 0.99 | | 1.08 | | .139 | 1.04 | 0.99 | | 1.08 | | .124 |
| *APOE4* status | 2.01 | 0.40 | | 1.00 | | **< .001** | 1.83 | 1.33 | | 2.50 | | **< .001** | 1.79 | 1.30 | | 2.45 | | **< .001** |
| GDS | 0.98 | -0.12 | | 0.08 | | .703 | 0.95 | 0.85 | | 1.06 | | .349 | 0.95 | 0.85 | | 1.06 | | .385 |
| GAS | 1.04 | -0.05 | | 0.12 | | .373 | 1.01 | 0.93 | | 1.10 | | .753 | 1.01 | 0.93 | | 1.10 | | .811 |
| Neuroticism | .99 | -0.04 | | 0.01 | | .349 | 0.99 | 0.96 | | 1.02 | | .357 | 0.99 | 0.96 | | 1.02 | | .378 |
| Openness | .97 | -0.055 | | 0 | | .053 | 0.97 | 0.95 | | 1.00 | | .072 | 0.97 | 0.95 | | 1.00 | | .072 |
| Consciousness | .99 | -0.032 | | 0.021 | | .680 | 0.99 | 0.96 | | 1.02 | | .573 | 0.99 | 0.97 | | 1.02 | | .613 |

Note: GDS = Geriatric Depression Scale; GAS = Goldberg Anxiety Scale; Neuroticism, Contentiousness and Openness scores are captured via the NEO-Five Factor Inventory.
