# Supplementary material for: Longitudinal changes in participant and informant reports of subjective cognitive complaints are associated with dementia risk
Source: Front Aging Neurosci. 2023 Feb 20;15:1044807. doi: 10.3389/fnagi.2023.1044807 (PMC9987247; doi:10.3389/fnagi.2023.1044807)
Supplement: Supplementary file 3 [file Table_3.docx]

**Supplementary Table 3.** Cause-specific hazard model accounting for the competing risk of death to predict incident dementia over 10 years for participants’ SCC intercept and slope and informants’ SCC intercept and slope, controlling for participants’ baseline demographics, *APOE4* carrier status, mood, and personality.

|  |  | | 95% CI | |  | |
| --- | --- | --- | --- | --- | --- | --- |
| **†Predictors** | HR | LL | | UL | | *p* |
| †Participant SCC intercept | 1.01 | 0.84 | | 1.21 | | .951 |
| †Participant SCC slope | 1.09 | 0.94 | | 1.27 | | .271 |
| †Informant SCC intercept | 1.61 | 1.31 | | 1.96 | | **< .001** |
| †Informant SCC slope | 1.38 | 1.14 | | 1.67 | | **< .001** |
| Age | 1.12 | 1.08 | | 1.16 | | **< .001** |
| Sex | 1.01 | 0.71 | | 1.42 | | .969 |
| Education | 1.04 | 0.99 | | 1.01 | | .112 |
| *APOE4* status | 1.95 | 1.39 | | 2.74 | | **< .001** |
| GDS | 0.95 | 0.84 | | 1.06 | | .342 |
| GAS | 1.02 | 0.93 | | 1.12 | | .687 |
| Neuroticism | 0.99 | 0.96 | | 1.02 | | .403 |
| Openness | 0.97 | 0.94 | | 1.00 | | **.041** |
| Conscientiousness | 0.99 | 0.97 | | 1.02 | | .720 |

Note: Censoring was specified on the date of death or at the end of follow-up/participant drop-out. GDS = Geriatric Depression Scale; GAS = Goldberg Anxiety Scale; Neuroticism, Contentiousness and Openness scores are captured via the NEO-Five Factor Inventory. †SCC intercept and slope for participants and informants are standardized against the sample average.
