# Supplementary material for: Longitudinal changes in participant and informant reports of subjective cognitive complaints are associated with dementia risk
Source: Front Aging Neurosci. 2023 Feb 20;15:1044807. doi: 10.3389/fnagi.2023.1044807 (PMC9987247; doi:10.3389/fnagi.2023.1044807)
Supplement: Supplementary file 4 [file Table_4.docx]

**Supplementary Table 4.** Results of Cox proportional hazard regression models using Wave 4 (6-year follow-up) as baseline to predict incident dementia over 4 years for participants’ SCC intercept and slope and informants’ SCC intercept and slope, controlling for participants’ baseline demographics, *APOE4* carrier status, mood, and personality.

Note: GDS = Geriatric Depression Scale; GAS = Goldberg Anxiety Scale; Neuroticism, Contentiousness and Openness scores are captured via the NEO-Five Factor Inventory. †SCC intercept and slope for participants and informants are standardized against the sample average.

|  |  | | 95% CI | |  | |
| --- | --- | --- | --- | --- | --- | --- |
| **†Predictors** | HR | LL | | UL | | *p* |
| †Participant SCC intercept | 1.10 | 0.86 | | 1.41 | | .442 |
| †Participant SCC slope | 1.12 | 0.92 | | 1.37 | | .272 |
| †Informant SCC intercept | 1.35 | 1.05 | | 1.74 | | **.019** |
| †Informant SCC slope | 1.23 | 0.95 | | 1.60 | | .113 |
| Age | 1.10 | 1.06 | | 1.15 | | **< .001** |
| Sex | 1.05 | 0.66 | | 1.67 | | .833 |
| Education | 1.05 | 0.99 | | 1.11 | | .140 |
| *APOE4* status | 2.01 | 1.33 | | 3.04 | | **.001** |
| GDS | 0.97 | 0.84 | | 1.13 | | .728 |
| GAS | 1.06 | 0.96 | | 1.17 | | .236 |
| Neuroticism | 0.97 | 0.94 | | 1.01 | | .204 |
| Openness | 0.97 | 0.93 | | 1.00 | | .059 |
| Conscientiousness | 1.00 | 0.97 | | 1.04 | | .903 |
