# Supplementary material for: Longitudinal changes in participant and informant reports of subjective cognitive complaints are associated with dementia risk
Source: Front Aging Neurosci. 2023 Feb 20;15:1044807. doi: 10.3389/fnagi.2023.1044807 (PMC9987247; doi:10.3389/fnagi.2023.1044807)
Supplement: Supplementary file 5 [file Table_5.docx]

**Supplementary Table 5.** Results of Cox proportional hazard regression models controlling for participants’ baseline MCI status (yes/no) predicting incident dementia over 10 years for participants’ SCC intercept and slope and informants’ SCC intercept and slope, controlling for participants’ baseline demographics, *APOE4* carrier status, mood, and personality.

|  |  | | 95% CI | |  | |
| --- | --- | --- | --- | --- | --- | --- |
| †Predictors | HR | LL | | UL | | *p* |
| †Participant SCC intercept | 1.02 | 0.86 | | 1.22 | | .811 |
| †Participant SCC slope | 1.09 | 0.92 | | 1.28 | | .327 |
| †Informant SCC intercept | 1.42 | 1.15 | | 1.75 | | **.001** |
| †Informant SCC slope | 1.36 | 1.12 | | 1.65 | | **.002** |
| Age | 1.11 | 1.07 | | 1.15 | | **< .001** |
| Sex | 0.99 | 0.69 | | 1.42 | | .947 |
| Education | 1.05 | 1.00 | | 1.10 | | .058 |
| *APOE4* status | 1.75 | 1.25 | | 2.45 | | **.001** |
| GDS | 0.95 | 0.84 | | 1.07 | | .396 |
| GAS | 1.00 | 0.91 | | 1.10 | | .990 |
| Neuroticism | 1.00 | 0.96 | | 1.03 | | .770 |
| Openness | 0.98 | 0.95 | | 1.01 | | .255 |
| Conscientiousness | 0.99 | 0.96 | | 1.02 | | .494 |
| MCI Status at Baseline (y/n) | 2.22 | 1.61 | | 3.05 | | **< .001** |

Note: GDS = Geriatric Depression Scale; GAS = Goldberg Anxiety Scale; Neuroticism, Contentiousness and Openness scores are captured via the NEO-Five Factor Inventory. †SCC intercept and slope for participants and informants are standardized against the sample average.
